# News and Coming Events

**Published:** 1908-02-08

**Authors:** 


### NEWS AND COMING EVENTS.

Mr. Edgar D. Shirwell has been appointed resident medical officer of the Royal Albert Hospital, Devonport.

Dr. J. A. Ormerod has been appointed to deliver the Harveian Oration before the Royal College of Physicians of London on October 19, Dr. Dudgeon the Horace Dobell Lectures, Dr. W. Pasteur the Bradshaw Lecture, and Dr. Leonard Guthrie the Fitzpatrick Lectures in November. Dr. R. T. Hewlett has been appointed the Milroy Lecturer for 1909.

The Murchison Scholarship, founded in 1880 to perpetuate the memory of Dr. Charles Murchison and awarded annually alternately by the University of Edinburgh and the Royal College of Physicians of London has been awarded to J. S. Edwards, M.B.Edin. The next examination for this scholarship will begin at the College on April 1. Any student in medicine is eligible as a candidate who has been a registered medical student during a period of not less than five nor more than seven years at a hospital in London or Edinburgh.

At the annual general meeting of Governors of the Italian Hospital, held on Saturday, January 25, the following resolution was unanimously carried :—"That the cordial thanks of the Governors be and are hereby tendered to the editors of the British and foreign Press and Press agencies for their continued courtesy and greatly valued help to the hospital during the past year."

Professor von Jacksch, of Vienna, has discovered a method of preventing the burning of the skin which so often results from the operation of the $x$-rays. Professor Jacksch has invented a silver shield, $\frac{1}{50}$ mm. in thickness, enveloped in a covering of cellulose. This placed over the portion of the body to be exposed to the action of the $x$-rays has been found to preserve the skin from any injury, whilst the influence of the rays upon the organs desired to be affected is in no way hindered.

Mr. E. F. Harrison has investigated *Combretum sundaicum*, a plant which is at present of interest on account of its alleged virtues as an anti-opium remedy. He reports in a recent number of the "Pharmaceutical Journal." The results obtained are unsatisfactory in that they are chiefly of a negative character. Neither the raw nor the roasted drug gives any indication of containing any principle of a definite nature, certainly nothing of an alkaloidal or crystalline nature.

Few American surgeons were better known, in name at least, to their English confrères than Nicholas Senn, whose death took place on January 2 last, at Chicago. Dr. Senn was 63 years of age, and was a native of Switzerland, though a naturalised American. He took his degree of M.D. at the University of Chicago in 1868, after a hard struggle, for his family was poor, and young Senn had to earn his living while studying his profession. For twenty years he practised in Milwaukee, rapidly winning a name on account of his intrepid courage, originality, and resource as a surgeon. In 1888, he was appointed Professor of Surgery at the Chicago College of Physicians and Surgeons, and to the Rush Medical College. He was a brilliant operator and his contributions to practical surgery have been among the most important placed before the profession during the last decade. In America his name was a household one, and elsewhere he was almost as well known. His death is a loss to surgery, and removes one of the most popular of the leaders of the profession in America.

The council of King's College have accepted the resignation of Dr. David Ferrier as physician of King's College Hospital. Dr. Ferrier will continue to hold the professorship of neuro-pathology in the medical school.

The annual general meeting of the Vice-Presidents and Governors of the Royal Maternity Charity will take place at 31 Finsbury Square, E.C., on February 11, 1908, at 3.30 p.m. precisely.

At the ordinary quarterly *Comitia* of the Royal College of Physicians of London, held on January 30, the President, Sir Richard Douglas Powell, occupying the chair, the following Fellows of the College were elected members of the Council :—Dr. G. E. Herman, Dr. D. W. Finlay, Dr. N. I. C. Tirard, and Dr. W. P. Herringham. Dr. T. D. Acland was elected an Examiner in Medicine in place of the late Dr. H. M. Murray, and Dr. Champneys was re-elected a representative of the College on the Central Midwives Board.

Professor James Bell Pettigrew, M.D., LL.D., Chandos Professor of Anatomy and Medicine in the University of St. Andrews, died on January 30 after a protracted illness. Professor Pettigrew, who had reached his 73rd year, was the oldest professor in the University. He was a voluminous writer on medical and scientific subjects. He was Croonian Lecturer to the Royal Society, president of the Royal Medical Society in 1860, and curator of the museum of the Royal College of Surgeons, Edinburgh, 1869. He was awarded the Godard Prize of the French Academy of Sciences in 1874.

The claim made on behalf of Sir J. T. Simpson as "the discoverer of chloroform" is severely criticised by Mr. J. P. Gilmour, of Glasgow, in a letter which appears in the *Pharmaceutical Journal.* The writer points out that Soubeiron, in 1831, discovered chloroform, his discovery being confirmed a year later by Dumas and Liebig. In 1838, Dr. Formby, of Liverpool, first prescribed it internally and established its value as an antispasmodic. In 1842, Dr. Glover, of Edinburgh, demonstrated its narcotic properties upon dogs, and in March 1847 Flourens proved that it was similar to ether as an anæsthetic. The same year Lawrence, of St. Bartholomew's Hospital, used it in surgical practice.

Two new wards containing twenty-seven beds were opened at the London Fever Hospital on Monday, January 27, by Lady Balfour of Burleigh, who with Lord Balfour made a tour of inspection of the wards. His lordship thought that what had been done reflected the highest possible credit upon the architect and builders. The Institution could look back on a useful career of more than one hundred years, it having been founded by the citizens of London at a meeting held at the Old Thatched House Tavern in 1801. After that time what were "Houses of Recovery" were established in various parts of London, the one of which they were the lineal descendants being in the neighbourhood of King's Cross, which institution in the 'forties contained some sixty beds, the site of which was acquired under Parliamentary powers by the Great Northern Railway, and upon part of which was built what is now known as King's Cross Station. The company subsequently bought the site of the present Hospital, which was then known as the "Kettle Field," and presented it to the Hospital with the sum of £26,000.

